# Afferents of the mouse linear nucleus

**DOI:** 10.1186/s13041-020-00602-8

**Published:** 2020-05-05

**Authors:** Huazheng Liang, George Paxinos

**Affiliations:** 1 Department of Neurology, Translational Research Institute of Brain and Brain-like Intelligence, Shanghai Fourth People’s Hospital Affiliated to Tongji University School of Medicine, 1878 North Sichuan Road, Hongkou District, Shanghai, 200081 China; 2 Neuroscience Research Australia, Sydney, NSW 2031 Australia

**Keywords:** linear nucleus, hindbrain, amygdala, paralemniscal nucleus, subcoeruleus nucleus, reticular formation, solitary nucleus

## Abstract

The linear nucleus (Li) was identified in 1978 from its projections to the cerebellum. However, there is no systematic study of its connections with other areas of the central nervous system possibly due to the challenge of injecting retrograde tracers into this nucleus. The present study examines its afferents from some nuclei involved in motor and cardiovascular control with anterograde tracer injections. BDA injections into the central amygdaloid nucleus result in labeled fibers to the ipsilateral Li. Bilateral projections with an ipsilateral dominance were observed after injections in a) jointly the paralemniscal nucleus, the noradrenergic group 7/ Köllike -Fuse nucleus/subcoeruleus nucleus, b) the gigantocellular reticular nucleus, c) and the solitary nucleus/the parvicellular/intermediate reticular nucleus. Retrogradely labeled neurons were observed in Li after BDA injections into all these nuclei except the central amygdaloid and the paralemniscal nuclei. Our results suggest that Li is involved in a variety of physiological functions apart from motor and balance control it may exert via its cerebellar projections.

## Introduction

The linear nucleus (Li) is found in the medullary reticular formation surrounding the middle segment of the compact part of the ambiguus nucleus (AmbC) in dorsal, medial and/or lateral aspects. It resembles an upside down L or a capital pi (Π) in Nissl stained or retrograde tracer labeled sections [37]. This part of the brain contains clusters of nuclei with diverse functions centered on speech, swallowing, cardiovascular and respiratory control and is expected to have different functional inputs from the rest of the brain.

The linear nucleus (Li) was first identified in 1978 by horseradish peroxidase retrograde labelling from rat cerebellar tactile regions [47]. Since then it has been confirmed that Li projects to the cerebellar cortex [9, 36], especially the paramedian lobule, crus2 [36], and deep cerebellar nuclei [38]. Given the strong projections of Li to the cerebellum which is important for movement control [7], it is expected that Li has a role in movement. Apart from cerebellar efferents, neither the efferents nor afferents of Li have been rigorously studied. However, incidental observations from earlier studies suggest several candidate structures becaue these structures provide inputs to the medullary reticular formation where Li is located. These include the central nucleus of the amygdala [14, 48], periaqueductal gray [45], the solitary nucleus (Sol) [2], Köllike-Fuse nucleus (KF) [17] and the lateral vestibular nucleus (LVe) [4]. Further studies using retrograde tracers also suggest that the Li projects to the raphe nuclei [13, 18], the lateral paragigantocellular reticular nucleus (LPGi) [28], the pedunculopontine tegemental nucleus (PPTg) [42], and the septal area [46]. The majority of the abovementioned nuclei have recognized roles in movement control, consistent with the likely role of the projections from Li to the cerebellum. Thus, some of these nuclei may provide relay signals to the cerebellum through Li. Some of these regions such as the amygdala, the solitary nucleus, raphe nuclei also have established roles in cardiovascular control and pain modulation, suggesting that Li may share these functions, if these regions are confirmed to project to the Li. Notably, each of these regions also project to the spinal cord to regulate these functions.

The Li can be identified by the expression of genes including slc17a7 (http://mouse.brain-map.org/gene/show/ 48802) and zic 1 (http://mouse.brain-map.org/experiment/show/72103843) relative to other parts of the medullary reticular formation. We have also shown in the adult rodent brain that seipin, a lipid metabolism related protein, is expressed in Li but is also expressed in surrounding regions including the gigantocellur reticular nucleus (Gi) [26]. HCN4, potassium/sodium hyperpolarization-activated cyclic nucleotide-gated channel 4, was first identified in the heart and brain [29]. It is involved in pacemaker activities in many brain areas including the cerebellum [6, 21]. The present study investigated whether HCN4 is expressed in Li.

The main objective of the study is to examine afferents to Li from the central amygdaloid nucleus, KF, the paralemniscal nucleus (PL) which is rostral and adjacent to KF, Gi, Sol and adjacent parvicellular/intermediate reticular nucleus (PCRt/IRt). All of these nuclei project to the spinal cord and are potentially involved in motor and cardiovascular control [22–25].

## Materials and methods

### Animals

C57BL/6 mice of 12-14 weeks of age, weighing 25-30 g were used and obtained from the Animal Resource Centre, Western Australia.

### Anterograde tracing

Mice were anesthetized by an intraperitoneal injection of ketamine (80 mg/kg) and xylazine (5 mg/kg) diluted in normal saline and placed in a mouse stereotaxic instrument (Kopf Instruments, Tujunga, CA, USA). The skull surface was exposed and a hole was made above the nuclei of interest at the following coordinates [35] (antero-posterior, medio-lateral, dorso-ventral coordinates, respectively: central amygdaloid nucleus- bregma: -0.82 to -1.58 mm, midline: +2.0 to +2.6 mm, surface: -4.25 to -4.75 mm; paralemniscal nucleus-Bregma: -4.16 to -4.72 mm, midline:+1.0 to +1.25 mm, surface: -2.175 to -2.5 mm; KF- Bregma:-4.83-5.33mm, midline:+1.0-1.75mm, surface:-3.0-4.25mm; solitary nucleus and parvicelluar/intermediate reticular nucleus:-6.59-7.07mm, midline: +0.75-1.5mm, surface: -2.75-3.5mm; gigantocellular reticular nuclei: Bregma:-5.63 to -7.19 mm, midline:0–1.25 mm, surface:-3.40 to -4.85 mm). The target areas were injected with 10-20 nl of biotinylated dextran amine (BDA) solution (10,000 MW, Thermofisher) using a 5 μl Hamilton syringe (Hamilton Company, Reno, NV, USA; the outer diameter is 0.711 mm). Five to ten mice were used for each nucleus. As a control group, two mice received BDA injections into the cisterna magna and others received BDA injections to adjacent brain nuclei. In all cases, the needle of the Hamilton syringe was left in place for 15 min after the injection and then the skin was sutured, topical antibiotic tetracycline was sprayed over the site of incision, buprenorphine was injected subcutaneously.

### Tissue preparation

The mice were anesthetized with a lethal dose of pentobarbital solution (0.1 ml, 200 mg/ml) 3 weeks after stereotaxic injections along with 5 mice that did not undergo tracer injections. They were then flushed with 30 ml of 0.9 % normal saline containing heparin (150 IU/mouse; Sigma) using a peristaltic pump (entry through the left ventricle of the heart), followed by 60 ml of 4 % paraformaldehyde (Sigma) (in 0.1 M phosphate buffer). The brain and spinal cord were removed and postfixed in 4 % paraformaldehyde for 2 h at 4 °C, and then transferred to 30% sucrose (in 0.1 M PB solution). After 48 h, brains were cut into 40 μm thick sections using a Leica CM 1950 cryostat. Every second section from the brain was used for anterograde tracing studies.

### Immunohistochemistry

Peroxidase immunohistochemistry for potassium/sodium hyperpolarization-activated cyclic nucleotide-gated channel 4 (HCN4) was undertaken on half of the brain sections of five mice that did not undergo surgery. Sections were washed and treated with 1 % H_2_O_2_ in 50 % ethanol before being transferred into 5 % goat serum in 0.1 M PB to block non-specific antigen binding sites. The sections were incubated in the primary anti-HCN4 (rabbit, 1:500, Alomone, APC-052) solution overnight and subsequently in the secondary antibody (biotinylated goat anti-rabbit IgG; Sigma, 1:200) for 2 h. The sections were then washed and transferred to an extravidin peroxi- dase solution (Sigma, 1:1000) for 2 h. Finally, the sections were incubated in a 3,3,-diaminobenzidine (DAB) reaction complex (Vector lab, Burlingame, CA, USA) until an optimal colour developed. At the end of the procedure, the sections were mounted and dehydrated before being coverslipped.

Sections from BDA-injected mice were washed and treated with 1% H_2_O_2_ in 50 % ethanol before being transferred to 5 % goat serum in 0.1 M PB to block the non-specific sites. After 2 h, sections were incubated in an extravidin peroxidase solution (Sigma, 1:1,000) for 2h. Finally, sections were stained using a DAB substrate reaction complex (Vector lab, Burlingame, CA, USA) until optimal colour developed. The rest of the procedures were the same as mentioned above.

### Nissl stain

This staining was used to identify the components of the linear nucleus on BDA-DAB stained sections. Sections were dehydrated for 30 seconds in each of the ethanol solution (50%, 70%, 95%, 100%) before being de-fatted in 95% ethanol with 5% acetic acid pre-warmed to 55-60^o^C for 15-20 minutes. They were then incubated in 0.2% cresyl violet solution pre-warmed to 55-60^o^C for 1-2 minutes. After rinsing with 70% ethanol for 30 seconds and 95% ethanol for 1 minute, sections were differentiated in 95% ethanol with 0.5% acetic acid for 10-15 minutes until optimal color was observed. Sections were then dehydrated in the gradient ethanol (70%, 95%, 100%, 5 minutes each) and cleared in xylene (10 minutes) before being coverslipped with the DPX mounting medium.

### Data analysis

Sections from BDA injected mice and immunohistochemistry were imaged using an Aperio scanner (ScanScope XT) under 20x magnification. Scanned images were opened with the Imagescope software and TIFF images were taken at 4x, 5x, 10x, and 20x magnifications. The brightness and contrast were adjusted in Adobe Photoshop 6 and organized in Adobe Illustrator CS6 before they were saved as TIFF images again.

## Results

### Immunohistochemical staining

**Fig. 1:**
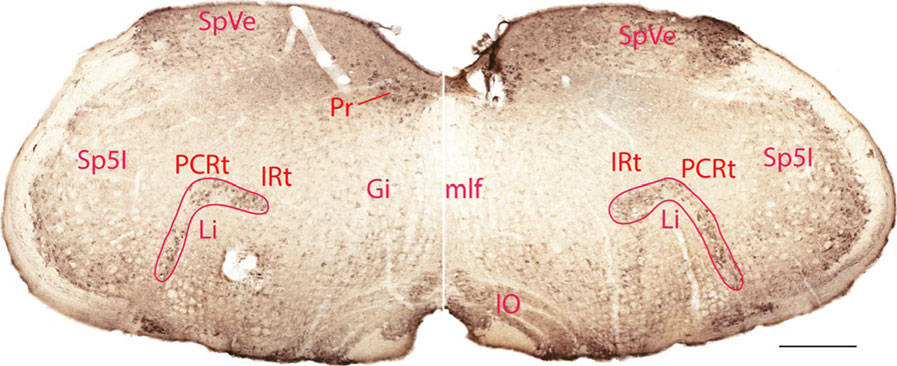
Immunohistochemical staining using an HCN4 antibody. HCN4 immunoreactivity in Li neurons is stronger than that of neurons in the rest of medullary reticular formation except some in the gigantocellular reticular nucleus. The scale bar=400 μm

The morphology of the linear nucleus in Nissl stain and various immunohistochemical stains were described by [9]. The present study found that HCN4 was diffusely located throughout the medulla and its expression in Li was more striking compared to the adjacent reticular formation (Fig. 1). In coronal sections, HCN4 positive neurons in Li formed an upside-down L shape. They were medial to the interpolar part of the spinal trigeminal nucleus (Sp5I), rostral to the lateral reticular nucleus (LRt), and clearly neighboring the compact part of the ambiguus nucleus.

### BDA injections to the central amygdaloid nucleus

**Fig. 2:**
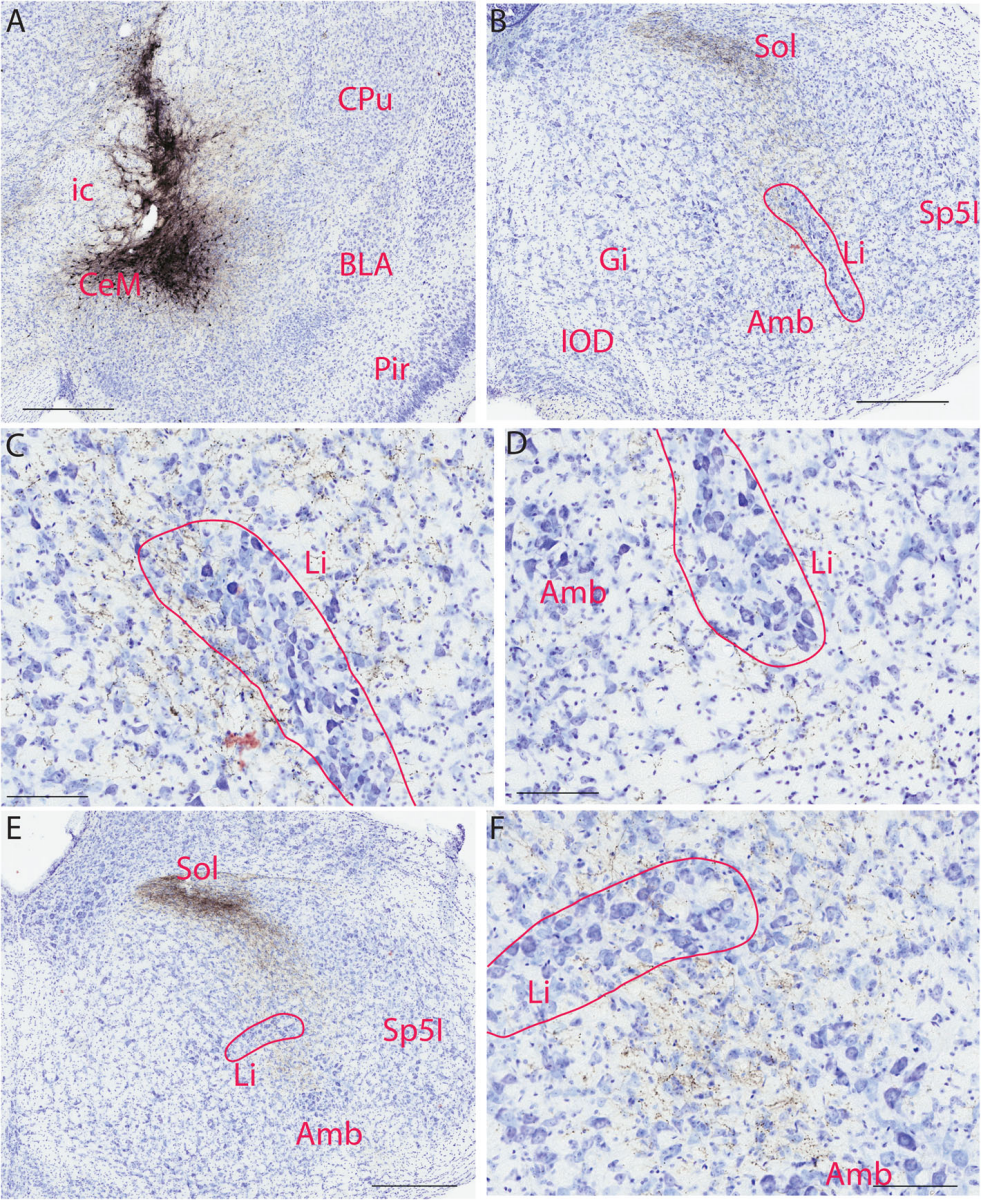
BDA labeled fibers in Li after injecting it to the medial part of the central amygdaloid nucleus (CeM). **a**. BDA injection site in CeM. **b**. Low magnification of a hindbrain section showing BDA labeled fiber bundle medioventral to Sol and fibers in Li. **c**. High magnification of **b** showing BDA labeled fibers terminating in the lateral limb of Li. **d**. High magnification of **b** showing BDA labeled fibers terminating in the ventral part of the lateral limb of Li. **e**. Low magnification of a hindbrain section showing BDA labeled fiber bundle medioventral to Sol and fibers terminating in the dorsal limb of Li. **f**. High magnification of **e** showing BDA labeled fibers terminating in the dorsal limb of Li. The scale bar in **a**, **b**, **e**=400 μm, the scale bar in **c**, **d**, **f**=100 μm

For five mice, BDA was aimed at the central amygdaloid nucleus, three were injected to the medial division of the central amygdaloid nucleus (CeM). One injection was confined to the anterior part of the basolateral amygdaloid nucleus (BLA), and one was located in the posterodorsal (MePD) and posteroventral (MePV) parts of the medial amygdaloid nucleus (unintended injection site). Injections to CeM (Fig. 2a) revealed a fiber bundle travelling from the medial part of Sol towards the medial border of Sp5I (Fig. 2b). Densely innervated area of the brain was the solitary nucleus, followed by the part of the intermediate reticular nucleus (IRt) and the parvicellular reticular nucleus (PCRt) adjacent to the limbs of Li. Most of Li is free of terminals from CeM. Some of the fibers bent ventrally and reached the margins of Li rostrally. Part of IRt ventral to Li and AmbC are negative for fiber terminals. Fibers from the medial side terminated moderately to the dorsomedial part of the lateral limb of Li (Fig. 2c,d), whereas fibers from the lateral side terminated sparsely to the middle portion of the lateral limb of Li (Fig. 2e,f). A small number of fibers terminated in the ventral end of the lateral limb of Li after arriving via a small ventral fiber tract (Fig. 2b,d). At the level of the dorsal limb of Li, sparse fibers were observed both dorsal and ventral to the nucleus, terminating in the dorsal and ventral aspects of this nucleus, respectively (Fig. 2e,f). Injections to both BLA and MePD/MePV did not show labeled fibers in Li (data not shown).

### BDA injections to the paralemniscal nucleus

**Fig. 3:**
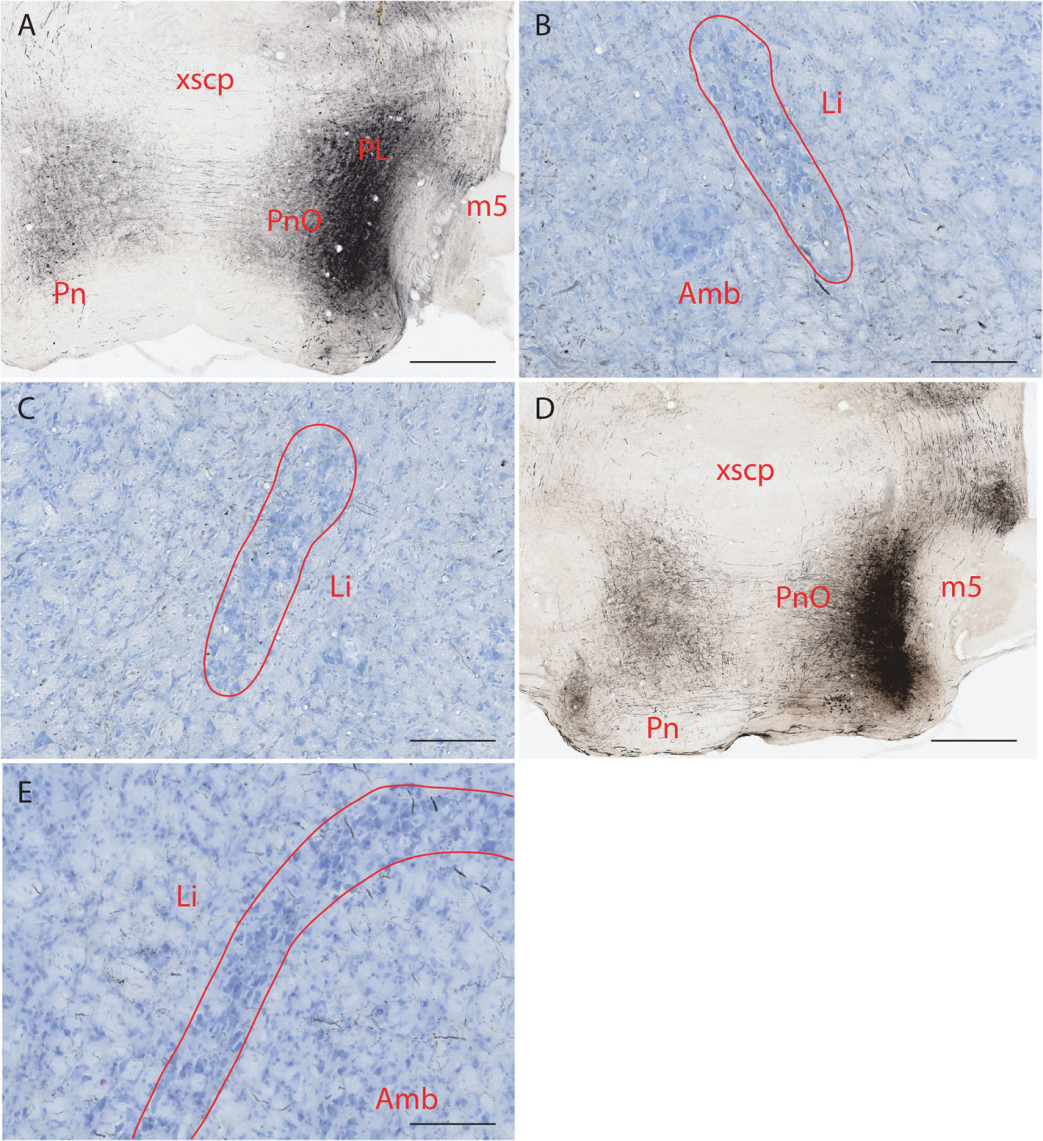
BDA labeled fibers in Li after injecting it to PL. **a**. BDA injection site in PL (note rubrospinal tract is encroached). **b**. BDA labeled fibers terminating in Li on the ipsilateral side. **c**. BDA labeled fibers terminating in contralateral Li. **d**. BDA injection site in caudal PL (note encroachment into PnO). **e**. BDA labeled fibers terminating in contralateral Li. The scale bar in **A** and **D**=400 μm, the scale bar in **b**, **c**, **e**=100 μm

Ten mice were used for PL injections but only seven were successfully target to the nucleus of lateral lemniscus. After injecting BDA to the rostral part of the PL (Fig. 3a), labeled fibers were seen to course through the medial part of the hindbrain lateral to or mingled with fibers in the medial longitudinal fasciculus, mainly on the ipsilateral side. Some of the fibers extended laterally towards LPGi and both the medial and lateral limbs of the Li. Sparse fibers were seen in Li. Some fibers were also seen ventral to Li (Fig. 3b). There is a possibility that some of them belong to the rubrospinal tract which might have been contaminated by BDA. On the contralateral side, labeled fibers were distributed similarly to those on the ipsilateral side but were far fewer. Some fibers were seen approaching Li from the area ventral and lateral to this nucleus (Fig. 3c). When BDA was injected to the caudal part of PL and the lateral part of oral pontine reticular nucleus (PnO) (Fig. 3d), labeled fibers were similarly distributed as those from the rostral PL. They were seen both medial and lateral to Li with a small number of these fibers terminating in Li. At the meeting point of the lateral and dorsal limbs of Li, there were a few thick fibers terminating in the area adjacent to Li, which were not seen in the rostral PL injections (Fig. 3e). Two injections to the rostral part of PnO revealed few fibers in Li (data not shown). One injection localized in the intermediate nucleus of the lateral lemniscus (ILL) did not result in labeled fibers in Li.

### BDA injections to Köllike-Fuse, supratrigeminal, and motor trigeminal nuclei

**Fig. 4:**
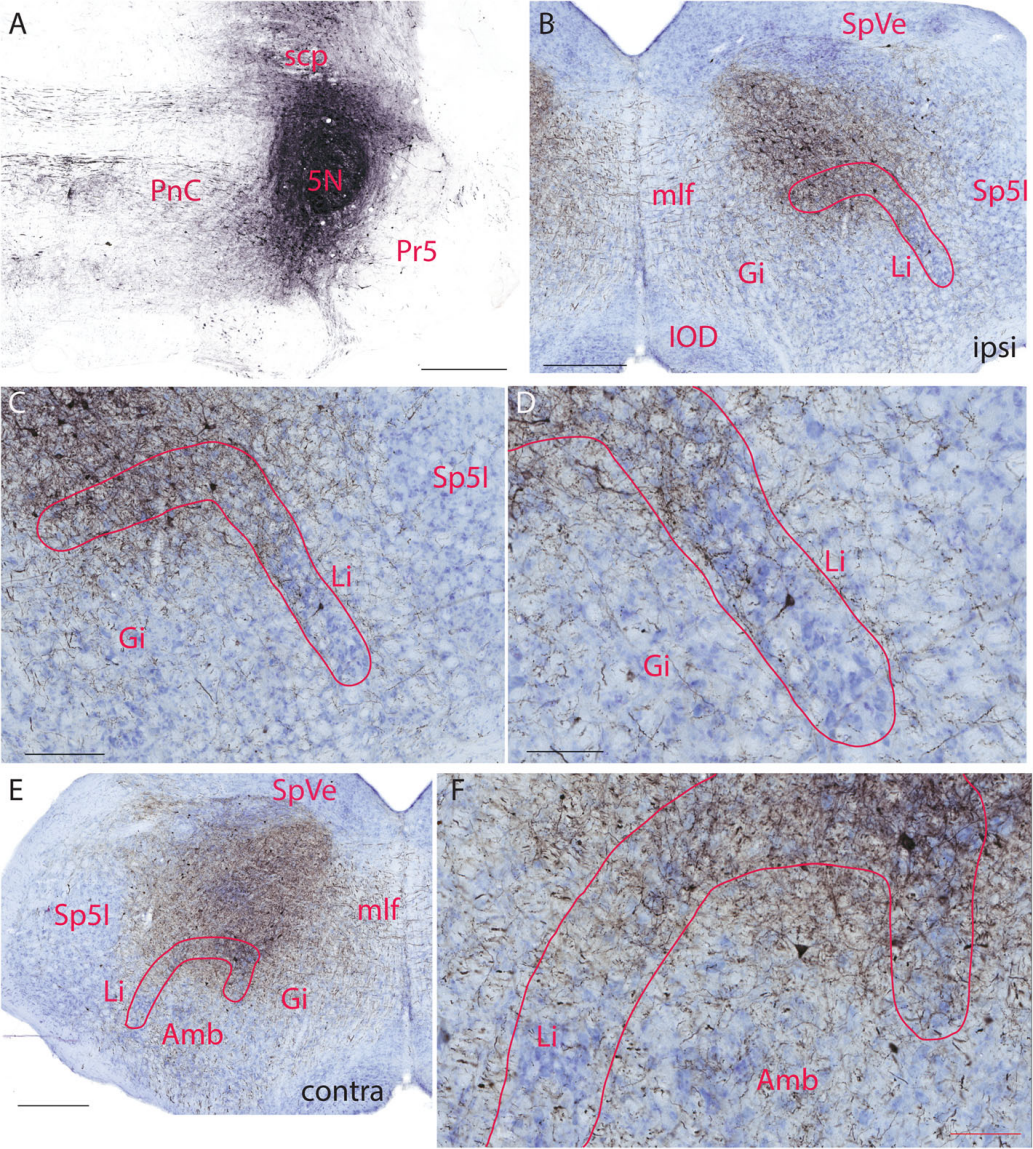
BDA labeled fibers in Li after injecting it to KF, Su5, and 5N. **a**. BDA injection site in KF, Su5, and 5N. **b**. Low magnification of a hindbrain section showing BDA labeled fibers and neurons in Li. **c**. High magnification of **b** showing BDA labeled neurons and fibers terminating in the limbs of Li on the ipsilateral side. **d**. A higher magnification of **c** showing a large number of BDA labeled fibers terminating in the limbs of Li (note a labeled neuron in the lateral limb of Li). **e**. Low magnification of a hindbrain section showing BDA labeled neurons and fibers terminating in the limbs of Li on the contralateral side. **f**. High magnification of **e** showing BDA labeled fibers terminating in the limbs of Li. The scale bar in **a**, **b**, **e**=400 μm, the scale bar in **c**=200 μm, **d**, **f**=100 μm

Nine mice were injected with BDA to target the Köllike-Fuse nucleus (KF) and supratrigeminal nucleus (Su5). However, none of them was injected into KF and Su5 alone without involving adjacent nuclei. When BDA injections encroached into the motor trigeminal nucleus (5N), Su5, and KF (Fig. 4a), a large number of labeled fibers was seen in the hindbrain ventral to Sol with some retrogradely labeled neurons on the ipsilateral side (Fig. 4b). Both the dorsal and lateral limbs of Li contained retrogradely labeled neurons and densely labeled fibers, especially in the dorsal limb. In the lateral part of the lateral limb of Li, densely labeled fibers were seen next to its lateral border (Fig. 4b-d). On the contralateral side, labeled fibers were as dense as those on the ipsilateral side and some fibers terminated in Amb, though less dense (Fig. 4e,f). In three mice, the injection site involved the parabrachial nuclei (PB) and the rostral part of the superior vestibular nucleus (SuVe) as well as KF, and 5N, there was a massive amount of labeled fibers in Li on both sides (data not shown).

### BDA injections to the gigantocellular reticular nucleus

**Fig. 5:**
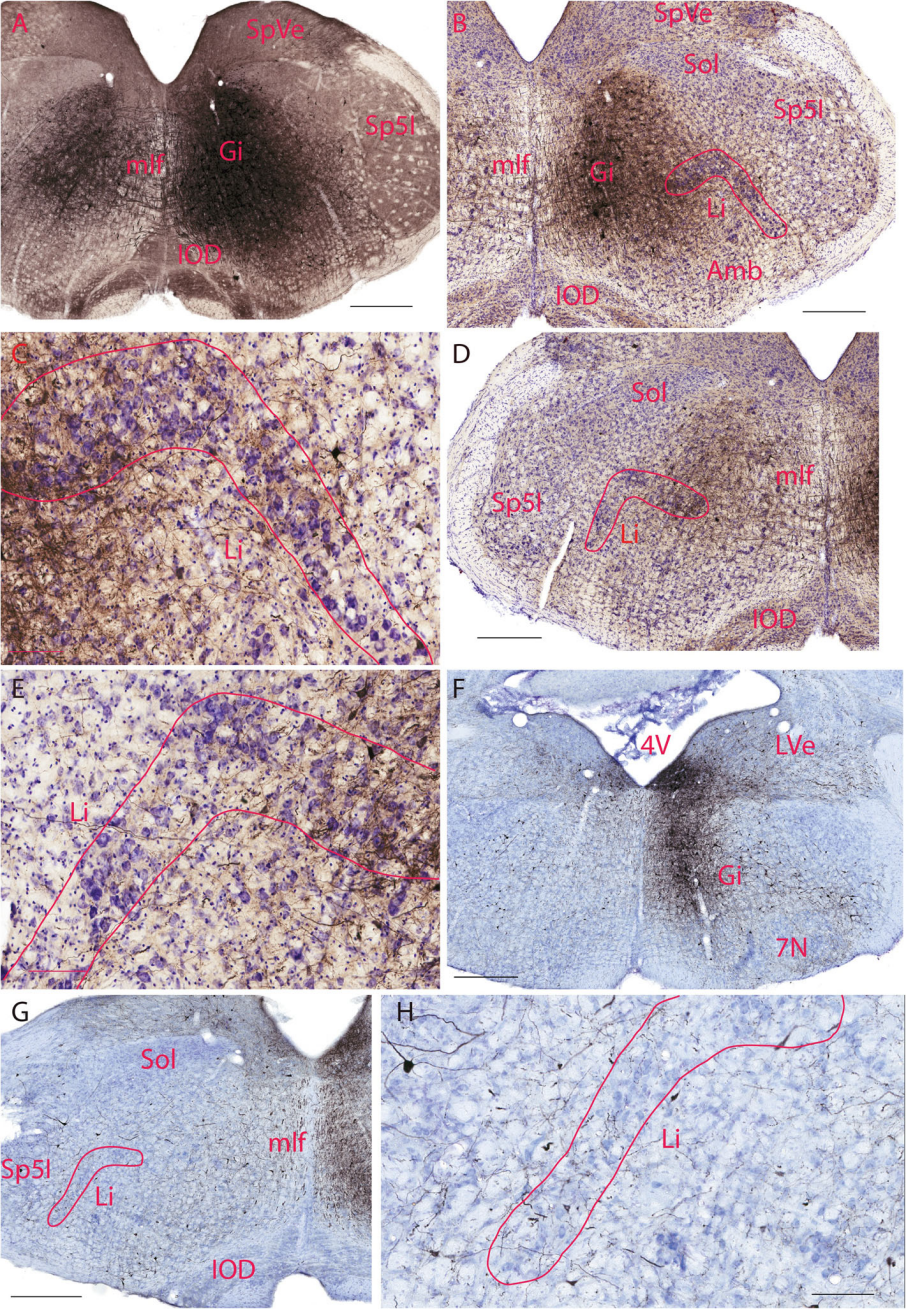
BDA labeled fibers in Li after injecting it to Gi. **a**. BDA injection site in Gi. **b**. Low magnification of a hindbrain section showing BDA labeled fibers in Li on the ipsilateral side. **c**. High magnification of **b** showing a large number of BDA labeled fibers terminating in the limbs of Li. **d**. Low magnification of a hindbrain section showing BDA labeled neurons and fibers terminating in the limbs of Li on the contralateral side. **e**. High magnification of **d** showing BDA labeled neurons and fibers terminating in the limbs of Li. **f**. Low magnification of a hindbrain section showing BDA injection site in Pr and DPGi. **g**. Low magnification of a hindbrain section showing BDA labeled neurons and fibers terminating in the limbs of Li on the contralateral side. **h**. High magnification of **g** showing BDA labeled neurons and fibers terminating in the limbs of Li. The scale bar in **a**, **b**, **d**, **f**, **g**=400 μm, the scale bar in **c**, **e**, **h**=100 μm

Twelve mice were injected with BDA into different components of Gi. There was only slight difference in BDA labeled fibers in Li after injections were made to different components of Gi (Fig. 5a-e). In general, densely labeled fibers from Gi terminated in all limbs of Li on both sides (Fig. 5b-e), with retrogradely labeled neurons seen occasionally (Fig. 5e). In two mice, BDA was injected into the rostral part of the dorsal paragigantocellular reticular nucleus (DPGi) and the adjacent prepositus nucleus (Pr), labeled fibers were again seen in Li on both sides, but the contralateral side had much fewer fibers than those from other parts of Gi (Fig 5f-h). In four mice, BDA was injected into either the alpha or the ventral part of Gi where serotonergic neurons are located, labeled fibers in Li were massive, leaving Amb as a relatively blank space (data not shown).

### BDA injections to the solitary nucleus and adjacent parvicellar reticular nucleus

**Fig. 6:**
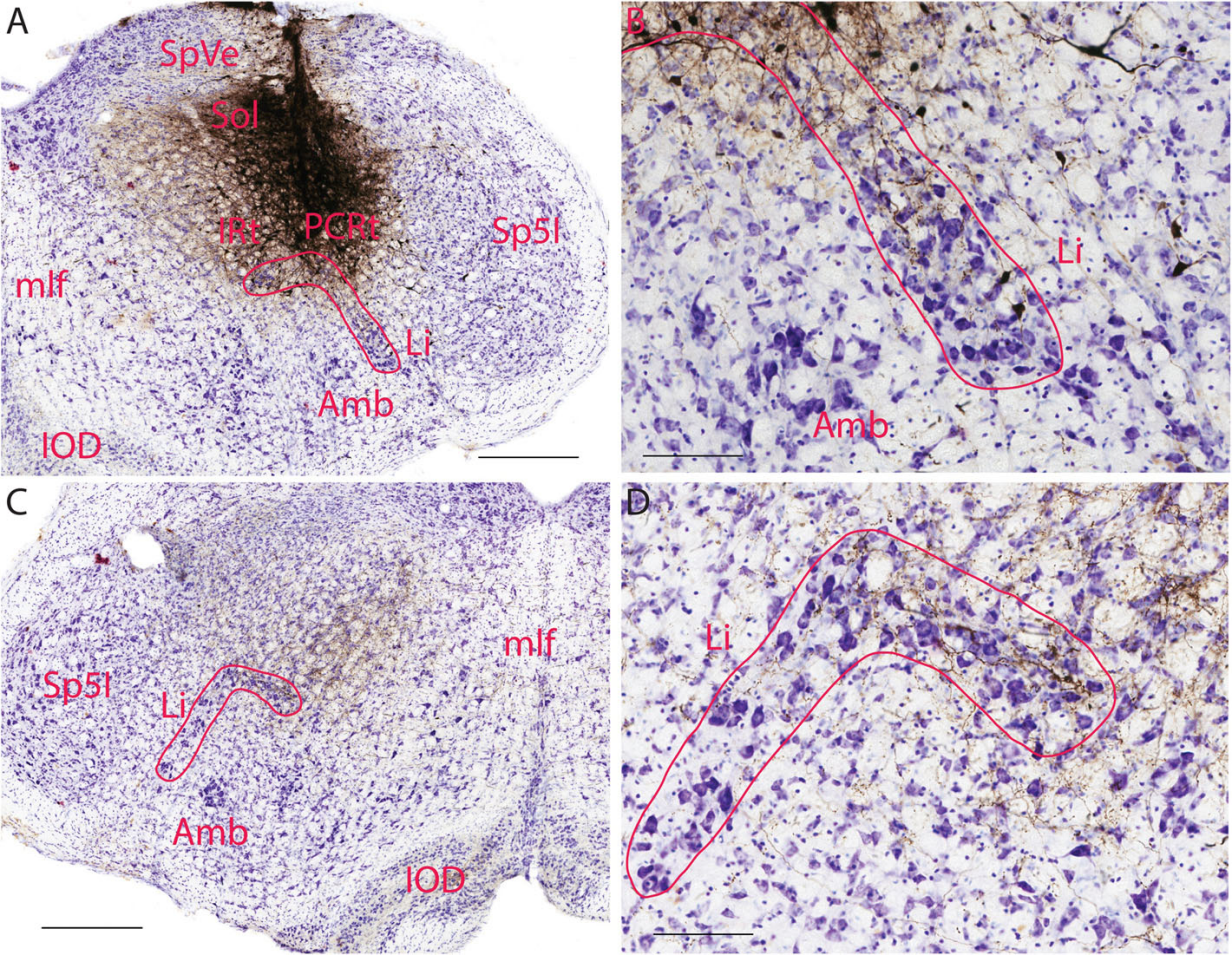
BDA labeled fibers in Li after injecting it to Sol and adjacent PCRt. **a**. BDA injection site in Sol and PCRt, as well as labeled neurons and fibers in Li. **b**. High magnification of **a** showing BDA labeled neurons and fibers terminating in Li on the ipsilateral side. **c**. Low magnification of a hindbrain section showing BDA labeled fibers terminating in Li on the contralateral side. **d**. High magnification of **c** showing BDA labeled fibers terminating in the limbs of Li. The scale bar in **a** and **c**=400 μm, the scale bar in **b** and **d**=100 μm

In nine mice, the target was the solitary nucleus (Sol) and the adjacent parvicelluar reticular nucleus (PCRt) (Fig. 6a). In seven mice, BDA injections into both nuclei with different degrees of encroachment into each nucleus revealed labeled fibers in Li on both sides (Fig. 6b-d). Fibers from the injection site extended towards Li on the ipsilateral side and terminated in its dorsal and lateral limbs (Fig. 6b). Among these fibers, a small number of labeled neurons were observed. When the injection site was more dorsal and further away from Li, similar fiber patterns were observed though fewer fibers were present in both the dorsal and lateral limbs of Li, especially when the injection site was confined to Sol (two mice, data not shown). On the contralateral side, labeled fibers crossed the midline and extended from the dorsal intermediate reticular nucleus (IRt) towards Li, terminating in both the dorsal and lateral limbs of Li with a predominance in the dorsal limb (Fig. 6c,d).

### BDA injections to the vestibular nuclei

In ten mice, the targets were the lateral (LVe) and the spinal vestibular (SpVe) nuclei. For six mice that received BDA injections into LVe, very few fibers were found in Li. Among four mice that received BDA injections into SpVe, one had BDA injection to the lateral part of SpVe and labeled fibers were occasionally seen. In the other three, BDA was injected into the central part of SpVe and few fibers en passage were found in Li (data not shown).

## Discussion

### Inputs to Li discovered in the present study

The projections to Li have remained largely unknown despite its discovery in 1978 [47]. We used an anterograde tracer to investigate its afferents from some nuclei that are associated with motor and cardiovascular control. For the first time, the present study reports that PL, KF and Su5, Gi, Sol and adjacent PCRt/IRt project to Li.

The present study found that BDA labeled fiber terminals were present in Li after injections were made to the central amygdaloid nucleus. Anterograde studies on efferents of the central amygdaloid nucleus found that fibers from this nucleus terminate in the lateral half of the medullary reticular formation, an area which harbours Li [14, 48]. Though the authors did not state this, the fibers are likely to terminate in Li, which is similar to our results. On the basis of sketchy data from the literature (see introduction), we hypothesized that CeM would be a source of afferents to Li. We found that CeM projects heavily to the Sol and IRt above Li, and only sparsely invests the medial and lateral margins of Li. Coincidently, Allen Institute for Brain Science database (http://connectivity.brain-map.org/bda-aav/experiment/thumbnails/113071071?popup=true&image_type=BDA_AAV) also has a mouse brain injected with BDA/AAV into this nucleus and the labeled fibers in Li are approximately the same as ours. More fiber terminals are present in the lateral limb of Li and relatively fewer terminals in the medial and dorsal limbs of Li (Supplementary Fig 1).

PL has been known for its projections to the spinal cord [19, 23, 34]. Its other efferents have not been reported previously. The present study found that it projected to Li, though the density of fibers was low. However, caution should be taken since this nucleus is a thin column of neurons and in close proximity to the rubrospinal tract. Contaminated fibers of the rubrospinal tract may reach Li from the ventral surface of the hindbrain.

An anterograde study showed descending fibers from the cuneiform nucleus (CnF) and KF to Amb and adjacent areas. This presents the possibility that Li is involved as well though the authors did not recognize this nucleus [17]. Our results from KF, Su5, 5N injections explicitly showed the presence of efferents from these nuclei to Li, suggesting that KF, Su5, 5N are very likely to project to Li. Furthermore, there are labeled neurons in Li after injections to the above nuclei, indicating mutual connections between them. Results from Allen Institute for Brain Science after BDA/AAV injection into KF alone showed similar findings as ours. Though the density of fiber terminals is not high, it is clear that these fibers are terminating in the same area as Li and adjacent nuclei (http://connectivity.brain-map.org/bda-aav/experiment/siv/100142301?imageId=102165855&imageType=BDA_AAV,SMI_32&initImage=BDA_AAV) (Supplementary Fig 2).

The present study found that the gigantocellular reticular nucleus (Gi) projected to Li and some labeled neurons were present in Li after BDA injections to it. This indicates that Gi has mutual projections to Li. In the cat, some neurons in the area rostral and dorsal to LRt were found to project to the lateral paragigantocellular reticular nucleus (LPGi). A portion of these neurons were continuous with those labeled in LPGi on the contralateral side [28], suggesting some of these neurons might belong to Li. In combination with previous findings, Li has bidirectional connections with Gi. Results from Allen Institute for Brain Science after BDA/AAV injection into Gi showed similar findings as ours. Heavily labeled fluorescent signals are present in the hindbrain except in Sol, the lateral limb of Li, and the trigeminal nucleus. Though the boundary between Li and the adjacent nuclei is not clearly defined, the medial limb of Li certainly has densely labeled fiber terminals on both sides. The lateral limb has less densely labeled fiber terminals (http://connectivity.brain-map.org/bda-aav/experiment/siv/100142687?imageId=102177701&imageType=BDA_AAV,SMI_32&initImage=BDA_AAV) (**Supplementary Fig****3****A**).

Our results showed that BDA injections to Sol and adjacent PCRt resulted in labeled fibers and a small number of labeled neurons in Li, indicating bidirectional projections between Sol/PCRt and Li. This confirms a prior study that suggested the presence of projections from Sol to areas adjacent to the ambiguus nucleus (Amb) [2]. Furthermore, there is likely a reciprocal projection between them given that another study reported that the area lateral to Amb projects to Sol [32]. Our results confirmed this finding by the presence of a small number of labeled neurons in Li after Sol injections. Results from Allen Institute for Brain Science are similar to ours (http://connectivity.brain-map.org/bda-aav/experiment/thumbnails/100144286?popup=true&image_type= BDA_AAV) (**Supplementary Fig****3****B**).

An anterograde study showed that fibers from LVe terminate not only in LRt but also the area dorsal to it [4]. These fibers may terminate in Li based on its anatomical proximity to LRt though the authors did not identify Li. Our results showed few labeled fiber terminals, especially after injections were made to SpVe. This might be due to the small size of BDA injections and the small number of injections in the vestibular nuclei which are relatively large rostrocaudally.

**Additional inputs to Li**

Anterograde tracer injections to the Edinger-Westphal (EW) and the tegmental area ventral to it resulted in labeled fiber terminals in the area lateral to Amb [27]. In another set of injections into the ventrolateral periaqueductal gray (VLPAG) and the tegmental area ventrolateral to it, abundant fiber terminals were found in the area surrounding Amb [27], suggesting these nuclei may project to Li.

The A5 noradrenergic region has been reported to project to the area dorsal and lateral to Amb. Ventrolateral to Amb, a small number of neurons were found to project to A5, indicating the presence of afferents from Li to A5 [3]. These findings suggest that Li is not a uniform nucleus but a complex structure with inputs and outputs processed by different compartments.

In an anterograde study, Teune et al reported that both the anterior interposed cerebellar (Int, especially the lateral part) and the lateral cerebellar (Lat) nuclei (especially the caudal part) project to the area lateral to Amb, which may correspond to Li [44], indicating that the cerebellum has mutual connections with Li given Li projects to the cerebellar cortex [9, 36]. In a study on afferents to Lat, it was found that some neurons dorsal to LRt projected to Lat. These cells formed a 90 degree right turned L shape [8], which is likely to correspond to neurons in Li. Based on previous findings from Teune et al, it might be possible that Li has bidirectional connections with Lat.

An anterograde study showed that the caudal ventrolateral medulla (CVL) projects to its adjacent Amb, as well as the area lateral to Amb [43], which is likely to include Li. In the database of Allen Institute for Brain Science, BDA/AAV was injected into IRt, PCRt, and Amb and labeled fibers were found in Li and adjacent nuclei on the contralateral side. This is similar to our findings (http://connectivity.brain-map.org/bda-aav/experiment/ thumbnails/100144286? popup=true&image_type=BDA_AAV).

Available results from the Allen Institute for Brain Science portal also show that many brain areas project to Li. Apart from the nuclei reported in the present study, other nuclei include the cuneate nucleus (Cu), facial motor nucleus (7N), principal sensory trigeminal nucleus (Pr5), interploar and caudal parts of the spinal trigeminal nucleus (Sp5I, Sp5C), caudal pontine reticular nucleus (PnC), intermediate reticular nucleus (IRt), primary and secondary motor cortex (M1, M2), lateral reticular nucleus (LRt), lateral hypothalamic area (LHA), superior colliculus (SC), mesencephalic reticular formation (mRt), periaqueduct gray (PAG), ventral tegmental area (VTA), zona incerta (ZI), motor trigeminal nucleus (5N), gracile nucleus (Gr), hypoglossal nucleus (12N), inferior olive (IO), anterior and posterior hypothalamic nuclei, paraventricular hypothalamic nucleus, dorsomedial nucleus of the hypothalamus (DM),ventral part of the tuberomammillary nucleus (VTM), interpeduncular nucleus (IP), dorsal motor nucleus of vagus (10N), primary visual area (V1), supratrigeminal nucleus (Su5), raphe obscurus nucleus (ROb), reticular part of the substantia nigra (SNR), and medial vestibular nucleus (MVe) also project to Li. These results need to be interpreted with caution since some injection sites are large and involve a few nuclei adjacent to the target.

### Genetic and immunohistochemical studies

The present study showed that Li has extremely dense HCN4 staining compared with adjacent brain areas. This has not been reported previously. In an *in situ hybridization* study, HCN4 gene was found to be moderately expressed in LRt and adjacent Gi, and weakly expressed in the rest of the hindbrain reticular formation [31]. This suggests that the level of mRNA is not always parallel to that of the protein. In an immunohistochemical study using a home-made HCN4 antibody, no cell bodies were observed in the hindbrain reticular formation [33]. The present study used a commercial antibody and the specificity has been confirmed. It is very likely that the signal in our study is real.

### Functional significance

Li receives afferents from a variety of nuclei, suggesting that it plays a complex role in diverse activities.

Transneuronal rabies virus injections to the pterygopalatine ganglion revealed a small number of neurons in the area dorsolateral to Amb, suggesting these neurons might belong to Li [41]. The pterygopalatine ganglion is one of the 4 parasympathetic ganglia of the head and neck and its axons innervate the lacrimal glands and the nasal mucosa, suggesting the role of Li in parasympathetic control.

In the brain, the central amygdaloid nucleus is well known for its role in regulating emotional response like fear and anxiety after gathering sensory information from various sources [16, 30]. The paralemniscal nucleus has been known as an important relay for startle response to acoustic, tactile, and vestibular stimuli [11, 39]. Therefore, projections from the PL and CeM to Li and subsequently to the cerebellum might lead to the maintenance of balance while completing the reflexive behavior. A7, the Köllike -Fuse and the subcoeruleus nuclei are known for the presence of noradrenalin in them and their projections to the spinal cord [1, 5, 12, 20]. Some of their fibers terminate in the intermediolateral column [23] and the ventromedial gray matter [15, 40]. Their projections to Li may suggest their involvement in sympathetic and movement control.

The solitary nucleus plays an important role in processing visceral information from various sources and transmitting to nuclei in the forebrain, hindbrain and the spinal cord [10]. It recruits neural pathways to produce appropriate cardiorespiratory responses [49]. Its projections to Li suggest the potential involvement of Li in cardiorespiratory control.

Li has extensive connections with the cerebellum and the hindbrain reticular formation which in turn has substantial projections to the spinal cord. These possibly confer to Li a role in balance control, coordination, and locomotion. Based on the continuity of Li with LRt, it is very likely that Li is an integration center in the caudal hindbrain processing information from multiple systems.

## Supplementary information

**Additional file 1: Figure S1.** BDA/AAV injections to the medial part of the central amygdaloid nucleus (CeM) from Allen Institute for Brain Science website. **A**. Dash line circled area is the injection site. **B**. Fluorescent fiber terminals in Li. Both green and red fiber terminals are present in the lateral limb of Li, and a smaller number of fiber terminals in the medial limb of Li.

**Additional file 2: Figure S2.** BDA/AAV injections to the Köllike -Fuse nucleus (KF) from Allen Institute for Brain Science website. **A**. Dash line circled area is the injection site involving both KF and a portion of supratrigeminal nucleus (Su5). **B**. Fluorescent fiber terminals in Li. A small number of both green and red fiber terminals are present in the lateral limb of Li.

**Additional file 3: Figure S3.** BDA/AAV injections to the medullary reticular nuclei from Allen Institute for Brain Science website. **A**. BDA/AAV injections to the gigantocellular reticular nucleus (Gi). Dash line circled area is the injection site. A large number of fiber terminals are present in the contralateral Li, especially in its lateral limb. **B**. BDA/AAV injections to the parvicellular and intermediate reticular nuclei (PCRt/IRt). Dash line circled area is the injection site. Both green and red fiber terminals are present in the contralateral Li.

## Data Availability

Original data are available on reasonable request from the corresponding author.
