# Supplementary figures and images for: Afferents of the mouse linear nucleus
Source: Mol Brain. 2020 May 5;13:67. doi: 10.1186/s13041-020-00602-8 (PMC7201812; doi:10.1186/s13041-020-00602-8)

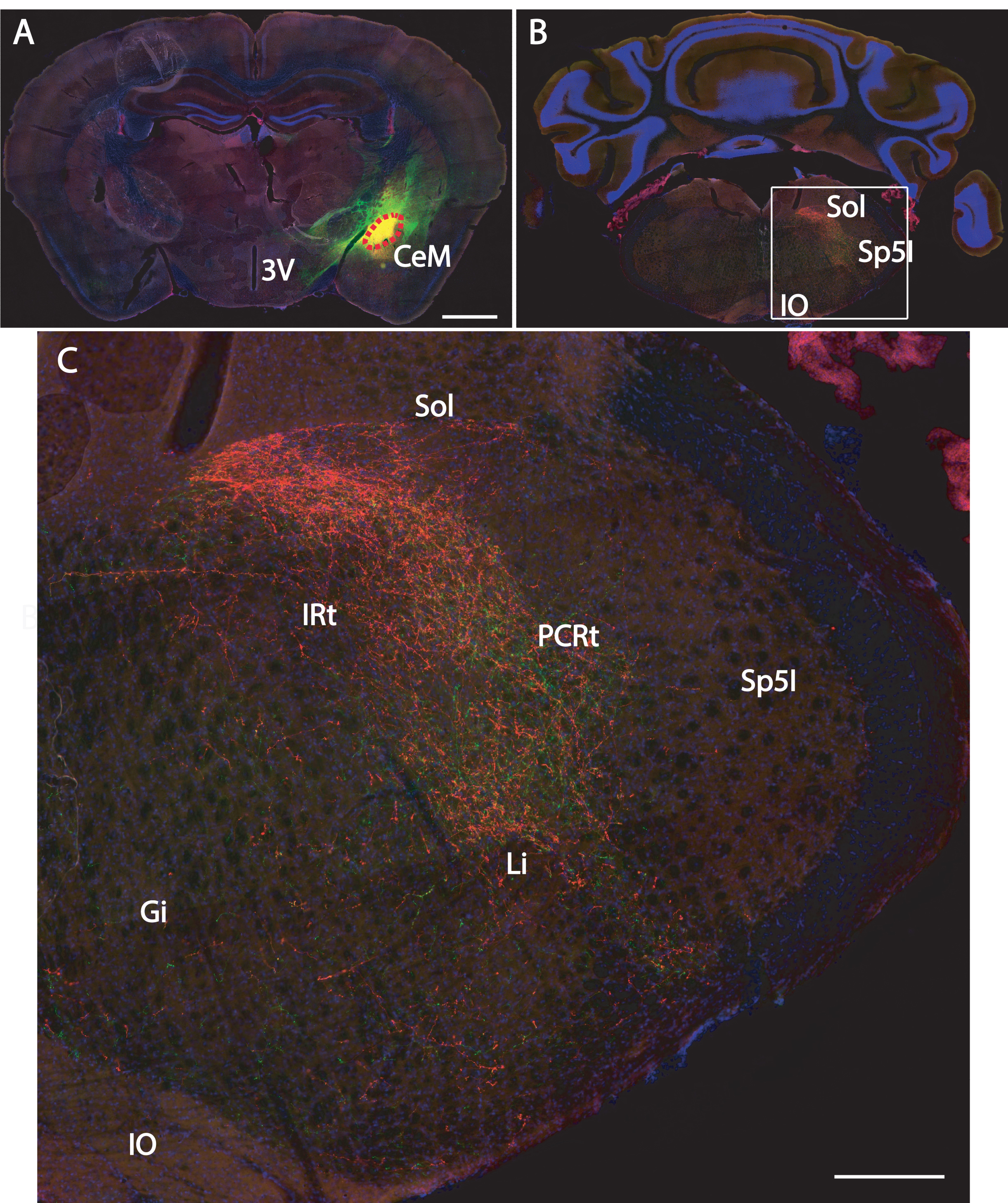

Supplement: Supplementary file 1 — Additional file 1: Figure S1. BDA/AAV injections to the medial part of the central amygdaloid nucleus (CeM) from Allen Institute for Brain Science website. A. Dash line circled area is the injection site. B. Fluorescent fiber terminals in Li. Both green and red fiber terminals are present in the lateral limb of Li, and a smaller number of fiber terminals in the medial limb of Li. [file 13041_2020_602_MOESM1_ESM.jpg]

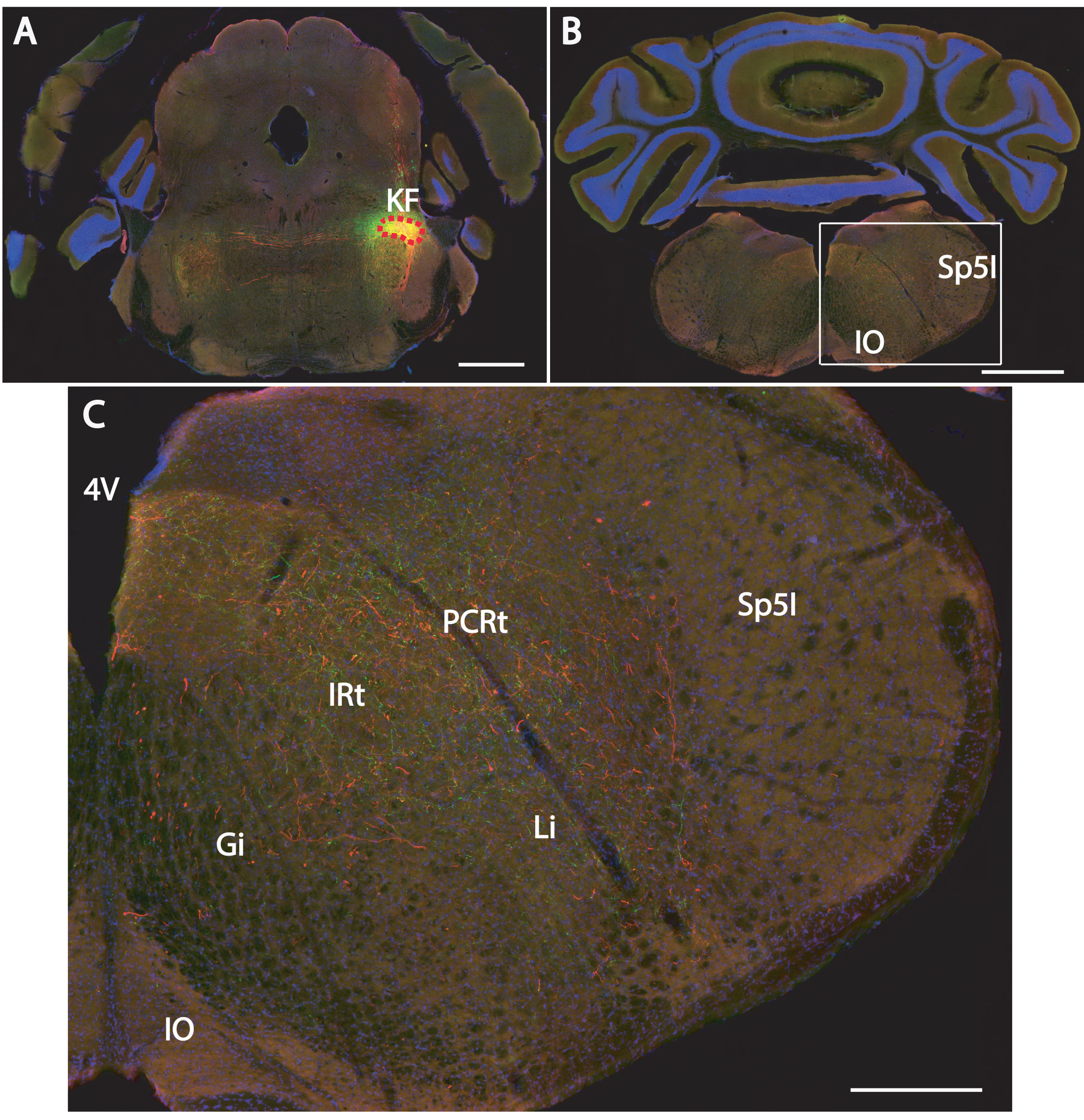

Supplement: Supplementary file 2 — Additional file 2: Figure S2. BDA/AAV injections to the Köllike -Fuse nucleus (KF) from Allen Institute for Brain Science website. A. Dash line circled area is the injection site involving both KF and a portion of supratrigeminal nucleus (Su5). B. Fluorescent fiber terminals in Li. A small number of both green and red fiber terminals are present in the lateral limb of Li. [file 13041_2020_602_MOESM2_ESM.jpg]

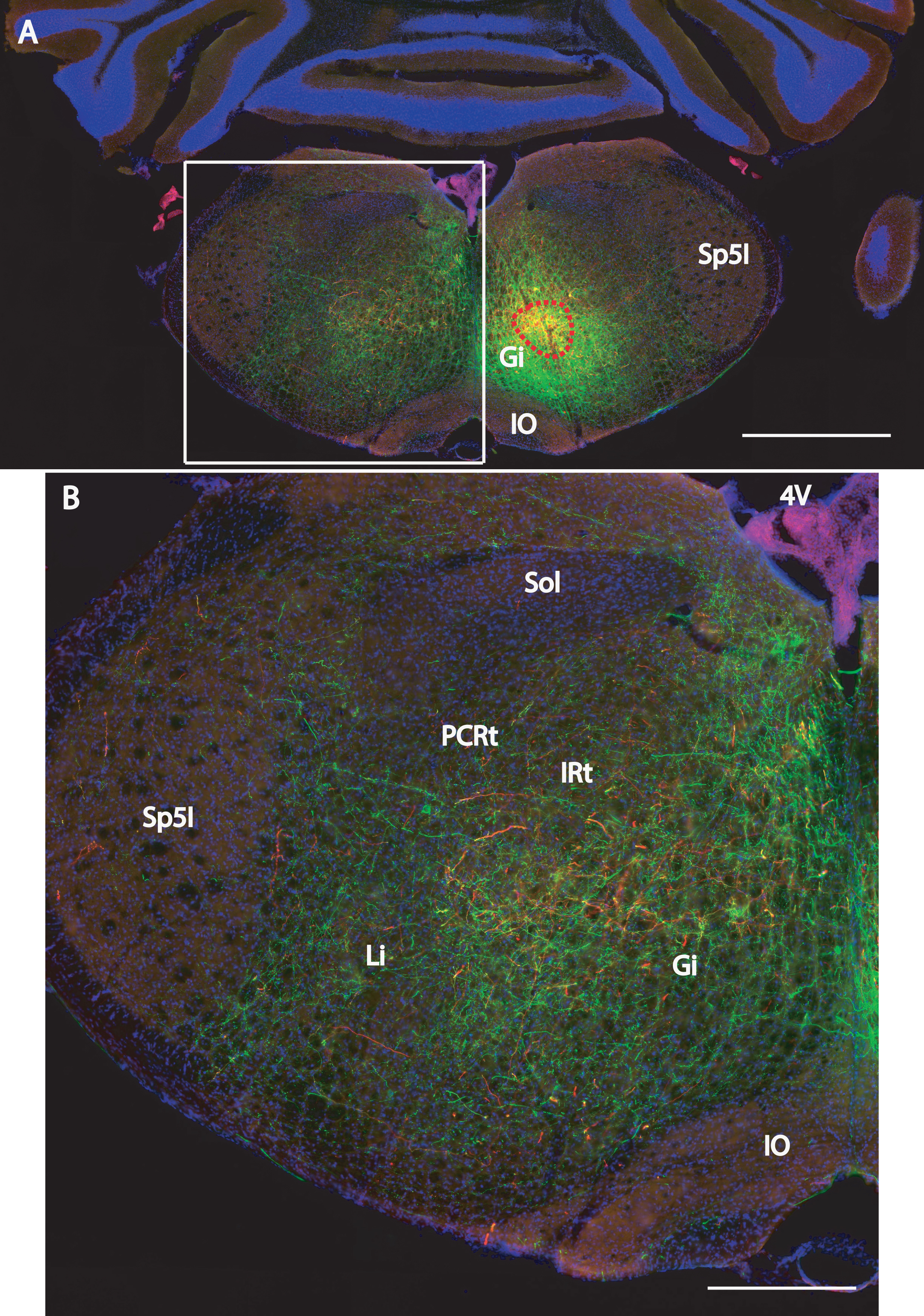

Supplement: Supplementary file 3 — Additional file 3: Figure S3. BDA/AAV injections to the medullary reticular nuclei from Allen Institute for Brain Science website. A. BDA/AAV injections to the gigantocellular reticular nucleus (Gi). Dash line circled area is the injection site. A large number of fiber terminals are present in the contralateral Li, especially in its lateral limb. B. BDA/AAV injections to the parvicellular and intermediate reticular nuclei (PCRt/IRt). Dash line circled area is the injection site. Both green and red fiber terminals are present in the contralateral Li. [file 13041_2020_602_MOESM3_ESM.jpg]
